# Trachoma

**Published:** 1889-11

**Authors:** E. P. Edwards


### CORRESPONDENCE.

### TRACHOMA.

*Ed. Daniel's Texas Medical Journal:*

The following treatment has yielded me much better results in granular lids, or trachoma, than the usual treatment with blue stone, nitrate silver, chlor., amm., etc.

| | | |
|---|---|---|
| ℞ | Hg. Bichlor. | gr. j. |
| | Aq. Destil | ℥ i. |

M. S. Five or six drops in the eyes three times a day. This solution is to be used at home by the patient. Twice a week, at my office, I apply to the everted lids, with a glass rod, because it is easily kept clean, and a brush is not,

| | | |
|---|---|---|
| ℞ | Hg. Bichlor. | grs. v. |
| | Aq. Destil | ℥ i. |

M. It is necessary to use cocaine pretty thoroughly before using the last solution, as it is quite painful. The first and second stages of granular lids are usually cured with this treatment in two to four months, and there does not result from the treatment the usual cicatricial tissue in the conjunctive, as from the treatment generally adopted, besides the time required to accomplish a cure is very much shortened. The pannus rapidly clears up under this medication, when it is dependent upon trachoma, and the treatment is not more painful than the usual one.

As granular lids is a very common ailment, I would be glad if the profession would try this treatment, and report their success with it; for, as I said in the beginning, it has yielded me better results than any other which I have tried, or seen tried.

One other thing: granular lids will be found quite often in even small children, oftener, in fact, than one would be led to suppose from some of the books, in proof of which it is only necessary to look for it, or watch the outdoor department of any ophthalmic hospital, and see the number of children who come with granular lids.

No treatment is curative in those cases in which the conjunctiva has already become cicatricial. Paliation is all there is for them. E. P. EDWARDS, M. D.

